# Biceps femoris long head morphology in youth competitive alpine skiers is associated with age, biological maturation and traumatic lower extremity injuries

**DOI:** 10.3389/fphys.2022.947419

**Published:** 2022-09-15

**Authors:** Daniel P. Fitze, Martino V. Franchi, Stefan Fröhlich, Walter O. Frey, Jörg Spörri

**Affiliations:** ^1^ Department of Orthopaedics, Sports Medical Research Group, Balgrist University Hospital, University of Zurich, Zurich, Switzerland; ^2^ Department of Orthopaedics, University Centre for Prevention and Sports Medicine, Balgrist University Hospital, University of Zurich, Zurich, Switzerland; ^3^ Department of Biomedical Sciences, Institute of Physiology, University of Padua, Padua, Italy

**Keywords:** muscle morphology, hamstrings, ultrasound imaging, injury prevention, alpine ski racing, youth athletes

## Abstract

Lower extremity injuries are common in competitive alpine skiers, and the knee and lower leg are often affected. The hamstring muscles, especially the biceps femoris long head (BFlh), can stabilize the knee and the hip and may counteract various adverse loading patterns during typical mechanisms leading to severe lower extremity injuries. The aim of the present study was to describe BFlh morphology in youth competitive alpine skiers in relation to sex, age and biological maturation and to investigate its association with the occurrence of traumatic lower extremity injuries in the upcoming season. 95 youth skiers underwent anthropometric measurements, maturity offset estimations and ultrasound assessment, followed by 12-months prospective injury surveillance. Unpaired t tests showed that the two sexes did not differ in BFlh morphology, including fascicle length (Lf), pennation angle (PA), muscle thickness (MT) and average anatomical cross-sectional area (ACSA_avg_). In contrast, U16 skiers had longer fascicles than U15 skiers (9.5 ± 1.3 cm vs 8.9 ± 1.3 cm, *p* < 0.05). Linear regression analyses revealed that maturity offset was associated with Lf (*R* ^2^ = 0.129, *p* < 0.001), MT (*R* ^2^ = 0.244, *p* < 0.001) and ACSA_avg_ (*R* ^2^ = 0.065, *p* = 0.007). No association was found between maturity offset and PA (*p* = 0.524). According to a binary logistic regression analysis, ACSA_avg_ was significantly associated with the occurrence of traumatic lower extremity injuries (Chi-square = 4.627, *p* = 0.031, R_Nagelkerke_^2^ = 0.064, Cohen f = 0.07). The present study showed that BFlh morphology is age- and biological maturation-dependent and that BFlh ACSA_avg_ can be considered a relevant modifiable variable associated with lower extremity injuries in youth competitive alpine skiers.

## Introduction

Competitive alpine skiing is a sport with a high risk of traumatic injuries (Jordan et al., 2017; Spörri et al., 2017). The lower extremities, especially the knee and the lower leg, are often affected (Fröhlich et al., 2021). This is also evident in youth competitive alpine skiers around growth spurts, where the knee and the lower leg are the body regions most affected by traumatic and overuse injuries (Schoeb et al., 2020). With respect to the causes of injury, skiers’ lack of physical fitness can be considered a key driver (Spörri et al., 2012). In addition, biological maturation (i.e., maturity offset) has been shown to be related to the occurrence and severity of traumatic injuries (Schoeb et al., 2020) and was found to have a moderate to strong relationship to hamstring peak force values measured during the execution of the Nordic Hamstring Exercise (NHE) (Franchi et al., 2019).

Regarding injury prevention, the hamstring muscles can act as knee stabilizers (MacWilliams et al., 1999) and have the potential to counteract the boot-induced ventral displacement of the tibia and internal rotation, as they typically occur during mechanisms leading to severe knee injuries and proximal intra-articular tibial fractures in skiers (Hasler and Hardegger, 1993; Bere et al., 2011). The hamstring muscles can also act as hip stabilizers in the case of traumatic hip injuries and may help to counteract the hip flexion moment during backward falls with the trunk bent forward, as they often occur in the immediate course of (proximal) tibia contusions or fractures (Stenroos et al., 2016). Moreover, the medial (semimembranosus and semitendinosus) and lateral (biceps femoris) hamstrings oppose external and internal rotation of the tibia, respectively (Maniar et al., 2022), which in turn may counteract rearfoot supination and rearfoot pronation during mechanisms leading to ankle sprains (Neumann, 2010). Finally, well-developed quadriceps and hamstring muscles may provide additional protection in high-energy impacts, such as those that frequently occur in ski-related femur fractures (Sterett and Krissoff, 1994).

With respect to traumatic knee injuries, the most frequent type and location of injury in youth competitive alpine skiers (Schoeb et al., 2020), the hamstrings (and in particular its lateral part, i.e., the long head of the biceps femoris (BFlh)) have a great potential to unload the anterior cruciate ligament (ACL), given its ability to counteract the internal rotation of the knee, its large capacity to generate muscle force, and its ability to generate sufficiently large posterior shear forces (Maniar et al., 2022). In addition, a recent study revealed that healthy individuals with a greater posterior-inferior directed slope of the lateral tibial plateau have increased BFlh volumes (Schmitz et al., 2017). During axial loading, a greater posterior-inferior directed slope of the lateral tibial plateau has in turn been associated with greater anterior tibial translation, greater internal tibial rotation (Beynnon et al., 2014), and increasing ACL force (Mclean et al., 2011). As both anterior tibial translation and internal tibial rotation are key components of mechanisms leading to severe knee injuries in alpine skiing and as the important functional role of the BFlh in counteracting these components is known, it is reasonable to assume that the morphology of the BFlh might be of particular interest for injury prevention.

Regarding the functional aspects of hamstring muscles typically assessed in competitive alpine skiers in the context of injury prevention, the hamstrings-to-quadriceps strength ratio (H/Q ratio) measured by an isokinetic dynamometer is probably the most well-known approach (Jordan et al., 2017; Spörri et al., 2017). This approach measures the maximal voluntary torque (MVT) during knee flexion and extension based on the hypothesis that strong hamstring muscles could prevent the anterior shift of the tibia relative to the femur during typical injury mechanisms (Jordan et al., 2017; Spörri et al., 2017). According to Johnson (1995), however, examining the peak-to-peak H/Q ratio alone is not sufficient. Based on preliminary results where seven athletes experienced an ACL injury after initial screening compared to 41 athletes who remained uninjured over the 3 years, the author proposes the assessment of the joint angle at which the hamstrings MVT results, as this was the only factor that differed significantly between the two groups. Moreover, given the timeframe in which ACL injuries typically occur (less than 60 ms) (Bere et al., 2011), it has also been proposed to complement the traditional H-Q ratio screening protocol with measurements of the rate of torque development (RTD) (Jordan et al., 2015).

In addition to assessing the functional aspects, an analysis of BFlh morphology based on ultrasound images could add a structural perspective. This could provide further valuable insights, as both joint angle-specific MVT and RTD can be associated with muscle architecture variables. Ultrasound imaging has been extensively used in both research and clinical settings to study the morphological and mechanical properties of muscle-tendon units (Sarto et al., 2021). Advanced ultrasound systems even allow the acquisition of panoramic images for muscle architecture (Noorkoiv et al., 2010) and anatomical cross-sectional area (ACSA) (Scott et al., 2012) assessments. For the assessment of muscle architecture, this is particularly advantageous for muscles with relatively long fascicles (e.g., BFlh), as otherwise a large part of the fascicle has to be extrapolated, leading to potential inaccuracies during data analysis (Franchi et al., 2020b). Moreover, in a cohort of youth competitive alpine skiers, panoramic ultrasound was recently shown to be a valid tool to measure ACSA and volume estimates for hamstring muscles when compared to MRI (Franchi et al., 2020a). However, to the best of our knowledge, there are currently no published data on BFlh architecture and the influence of BFlh morphology on the occurrence of traumatic injuries of the lower extremities in youth skiers.

Based on these considerations, the aims of the present study were twofold: 1) to describe BFlh morphology in youth competitive alpine skiers with respect to sex, age and maturity offset and 2) to investigate its association with the occurrence of traumatic injuries of the lower extremities in the upcoming season.

## Materials and methods

### Study design, participants and setting

The present study was designed as a cohort study with baseline measurements followed by 12-months prospective injury surveillance at 2-week intervals. 99 competitive alpine skiers voluntarily participated in the baseline measurements. All participants were recruited through announcements and information dissemination within the youth development structure of the Swiss National Skiing Association (Swiss-Ski). Eligible to participate were skiers who were members of certified regional performance centers (RLZ/RPC), i.e., the best skiers in their age group throughout Switzerland. The exclusion criteria were as follows: skiers should have not been enrolled in a back-to-sports journey after an injury and should not present systematic pathologies such as inflammatory arthritis. Based on these criteria, no participants were excluded. However, we acknowledge four dropouts during the 12-months prospective injury surveillance period, as they ended their sports career. Accordingly, a total of 95 youth competitive alpine skiers with complete datasets were included in the final analysis, of which 33 were female (mean age = 14.7 ± 0.6 years) and 62 were male (mean age = 14.9 ± 0.7 years). To investigate sex- and age-specific differences, the entire cohort was subdivided into a female and a male group, as well as into skiers under 16 years of age (U16) and skiers under 15 years of age (U15). The underlying study protocol was approved by the local ethics committee of the Canton of Zurich (KEK-ZH-NR: 2017-01395) and was conducted according to the ethical standards of the Declaration of Helsinki and national laws. All participants provided written informed consent. If they were younger than 14 years, their legal guardians signed instead.

### Anthropometric measures and maturity offset estimations

The anthropometric measures included the assessment of body mass using a body scale and body height using a measuring tape to calculate the body mass index (BMI). In addition, chronological age and sex were recorded. For the estimation of biological maturation, the noninvasive method of Mirwald et al. (2002) was used. This method has already been validated for use in youth competitive alpine skiers (Müller et al., 2015). The sex-specific Mirwald formula uses the leg length (calculated from body and sitting height) and the chronological age at the time of measurement to determine the time before or after the age of fastest growth, the so-called maturity offset. The maturity offset thus reflects the difference between the time of assessment and the time when the skier is expected to reach the maximum growth rate (negative values) or the time already exceeded since reaching the maximum growth rate (positive values).

### Ultrasound measurements

The ultrasound measurements were performed at the Swiss Centre for Musculoskeletal Imaging (SCMI). All ultrasound images were acquired by an experienced operator (MF) using an ultrasound device (Aixplorer Ultimate, SuperSonic Imagine, Aix-en-Provence, France). Study participants were instructed to lie prone on the massage bed with their ankles on the edge of the bed so that their feet could be kept in a neutral position and the hip and knee joints were extended. To compensate for body fluid shifts, the time between the positioning of the study participants and the image acquisition was at least 5 min, as proposed by (Perkisas et al., 2018).

An equivalent procedure for identifying and marking the region of interest (ROI), generating ultrasound images, and image analysis has been described in detail in Franchi and others (Franchi et al., 2020a; Franchi et al., 2020b). Briefly, the right posterior thigh of the study participants was first marked at 30, 40, 50, and 60% of the femur length (distance between the greater trochanter and distal end of the lateral femoral condyle) using a permanent marker. Subsequently, at each mark, the medial and lateral borders of the BFlh were identified and marked using transversal scans. These markers served as guidelines for generating longitudinal panoramic images.

**FIGURE 1:**
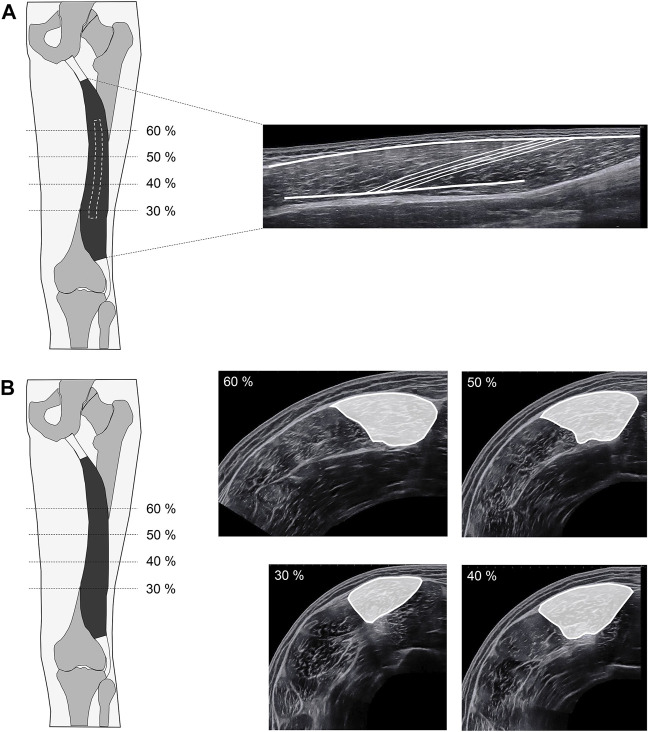
Exemplary ultrasound scans. **(A)** Longitudinal scan; **(B)** transversal scans.

For the measurement of muscle architecture, longitudinal panoramic images were generated using a 5 cm linear transducer (SuperLinear SL18-5, SuperSonic Imagine, Aix-en-Provence, France). This involved moving the transducer from the distal to the proximal myotendinous junction in a slow, controlled manner with low pressure on the underlying tissue. During image acquisition, the orientation of the transducer was adjusted to keep as many fascicles and the superficial and intermediate aponeurosis visible as possible (please see Figure 1A for a representative scan). For the measurement of anatomical cross-sectional areas (ACSA), transversal panoramic images at 30, 40, 50, and 60% marks were generated using a 4 cm linear transducer (SuperLinear SL10-2, SuperSonic Imagine, Aix-en-Provence, France). This also involved moving the transducer slowly, in a controlled manner and with low pressure from the lateral to the medial border of the BFlh. For all images, a sufficient amount of ultrasound gel was applied to the acquisition path as a conductive medium and to ensure uniform movement of the transducer.

Image analysis was performed by an experienced rater (DF) using image processing software (ImageJ, National Institutes of Health, Bethesda, MD). Figure 1A shows an example of a panoramic longitudinal scan including traced aponeuroses and fascicles. For each image, the superficial and intermediate aponeurosis and four fascicles were drawn. Muscle architecture and size measurements included fascicle length (Lf), pennation angle (PA) and muscle thickness (MT). For the statistical analysis, the respective four values for Lf, PA and MT were averaged. Figure 1B shows examples of panoramic transversal scans, including drawn ACSA of the BFlh at 30, 40, 50 and 60% of the femur length. For statistical analysis, ACSAs between 30 and 60% of the femur length were averaged (i.e., ACSA_avg_) to account for the regional differences in ACSA along the femur length.

### Injury surveillance

The Oslo Sports Trauma Research Centre (OSTRC) questionnaire on health problems was used for 12-months prospective injury surveillance (Clarsen et al., 2014). Self-reported data were collected and managed using the secure, web-based software platform REDCap®. The participants of the study were sent an e-mail with a personal web link to the questionnaire every second Monday. In addition, automatic reminder messages were mailed 2 days later. If the study participants did not reply within 3 days, they and their parents were asked to complete the questionnaire again by text message. The possibility of completing the questionnaire ended after 7 days. The self-reported health problems from the questionnaires were divided into three basic categories: illness, traumatic injury and overuse injury (Clarsen et al., 2014). Traumatic injuries were defined as those related to a clearly identifiable event (trauma), while no such triggering event could be identified for overuse injuries (Fuller et al., 2006). After completion of the 12-months prospective observation phase, all study participants were personally examined and retrospectively interviewed by an experienced sports physician (SF) to verify the accuracy of the OSTRC questionnaire data reported.

### Statistical analysis

Statistical analysis was performed using statistical software (SPSS Statistics 26, IBM, Armonk, United States). To verify the normality of the distribution of any metric data, the Kolmogorov‒Smirnov (KS) test, graphical techniques (i.e., histograms and quantile-quantile plots) and shape parameters (i.e., skewness and kurtosis coefficients) were used. Due to a slight departure from the distribution normality of the age variable (skewness and kurtosis values < 0.5 and < 1.2), all age-related statistical tests were backed up by bias-corrected accelerated (BCa) bootstrapping with 10,000 samples. In all other cases, standard parametric tests were applied.

Anthropometric measures and BFlh muscle morphology data are described as the mean ± SD and were tested for significant sex and age-group differences using unpaired sample t tests (*p* < 0.05). To assess the association of the four variables related to BFlh morphology with biological maturation (i.e., the predictor “maturity offset”), linear regression models were used. Finally, the association between BFlh morphology and the occurrence of traumatic injuries of the lower extremities was investigated by conducting a binary logistic regression analysis (backward LR method).

## Results

### Overview of anthropometric measures and maturity offset estimations in youth competitive alpine skiers

Table 1 shows an overview of anthropometric measures and maturity offset estimations of the participating youth skiers. Age did not differ significantly between the two sexes, but the group of U16 skiers was confirmed to be on average significantly older than the group of U15 skiers (*p* < 0.001). Regarding maturity offset, female skiers had higher values than male skiers (*p* < 0.001), and U16 skiers had higher values than U15 skiers (*p* < 0.01). Male skiers were on average taller than females (*p* < 0.01), and U16 skiers were on average taller than U15 skiers (*p* < 0.01). Body mass did not differ significantly between the sexes. However, U16 skiers were on average heavier than U15 skiers (*p* < 0.05). There was no significant difference in BMI between sexes or age groups.

**TABLE 1:** Overview of the participants at baseline.

	Overall (*n* = 95)	Female (*n* = 33)	Male (*n* = 62)	U16 (*n* = 37)	U15 (*n* = 58)
Age (y)	14.8 ± 0.6	14.7 ± 0.7	14.9 ± 0.5	15.4 ± 0.2	14.4 ± 0.3###
Maturity Offset (y)	1.2 ± 1.1	2.3 ± 0.6	0.6 ± 0.8***	1.5 ± 1.1	1.0 ± 1.1##
Body Height (cm)	166.6 ± 7.6	163.6 ± 5.8	168.2 ± 8.0**	169.1 ± 7.9	164.9 ± 7.0##
Body Mass (kg)	56.4 ± 9.1	55.5 ± 6.9	56.9 ± 10.1	59.1 ± 9.1	54.6 ± 8.8#
BMI (kg/m^2^)	20.2 ± 2.2	20.7 ± 2.1	20.0 ± 2.2	20.6 ± 2.1	20.0 ± 2.2

Data are expressed as mean ± SD., Level of significance based on unpaired sample t-tests backed-up by bias-corrected accelerated (BCa) bootstrapping with 10,000 samples: ** and *** refer to a significant between-sex difference at *p* < 0.01 and *p* < 0.001, respectively. ^#^, ^##^ and ^###^ refer to significant age-group differences at *p* < 0.05, *p* < 0.01 and *p* < 0.001, respectively. U16: skiers aged under 16 years; U15: skiers aged under 15 years; BMI: body mass index.

### Overview of the biceps femoris long head morphology in youth competitive alpine skiers

Table 2 shows an overview of the BFlh morphology. The two sexes did not differ significantly in the BFlh morphology variables Lf, PA, MT and ACSA_avg_. U16 skiers had, on average, a larger Lf than U15 skiers (*p* < 0.05), whereas PA, MT and ACSA_avg_ did not differ significantly between the age groups.

**TABLE 2:** Overview of the biceps femoris long head morphology at baseline.

	Overall (*n* = 95)	Female (*n* = 33)	Male (*n* = 62)	U16 (*n* = 37)	U15 (*n* = 58)
Lf (cm)	9.2 ± 1.3	9.2 ± 1.4	9.2 ± 1.3	9.5 ± 1.3	8.9 ± 1.3^*#*^
PA (°)	11.0 ± 2.3	11.0 ± 2.5	11.0 ± 2.3	11.2 ± 2.5	10.9 ± 2.3
MT (cm)	1.9 ± 0.3	1.9 ± 0.3	1.8 ± 0.3	1.9 ± 0.3	1.8 ± 0.3
ACSA_avg_ (cm^2^)	8.2 ± 1.5	7.8 ± 1.3	8.4 ± 1.6	8.5 ± 1.4	7.9 ± 1.6

Data are expressed as mean ± SD., Level of significance based on unpaired sample t-tests backedup by bias-corrected accelerated (BCa) bootstrapping with 10,000 samples: There were no significant differences between-sex differences at *p* < 0.05. # refers to a significant age-group difference at *p* < 0.05. U16: skiers aged under 16 years; U15: skiers aged under 15 years; Lf: fascicle length; PA: pennation angle; MT: muscle thickness; ACSA_avg_: average anatomical cross-sectional area.

### Associations between maturity offset and biceps femoris long head morphology

**FIGURE 2:**
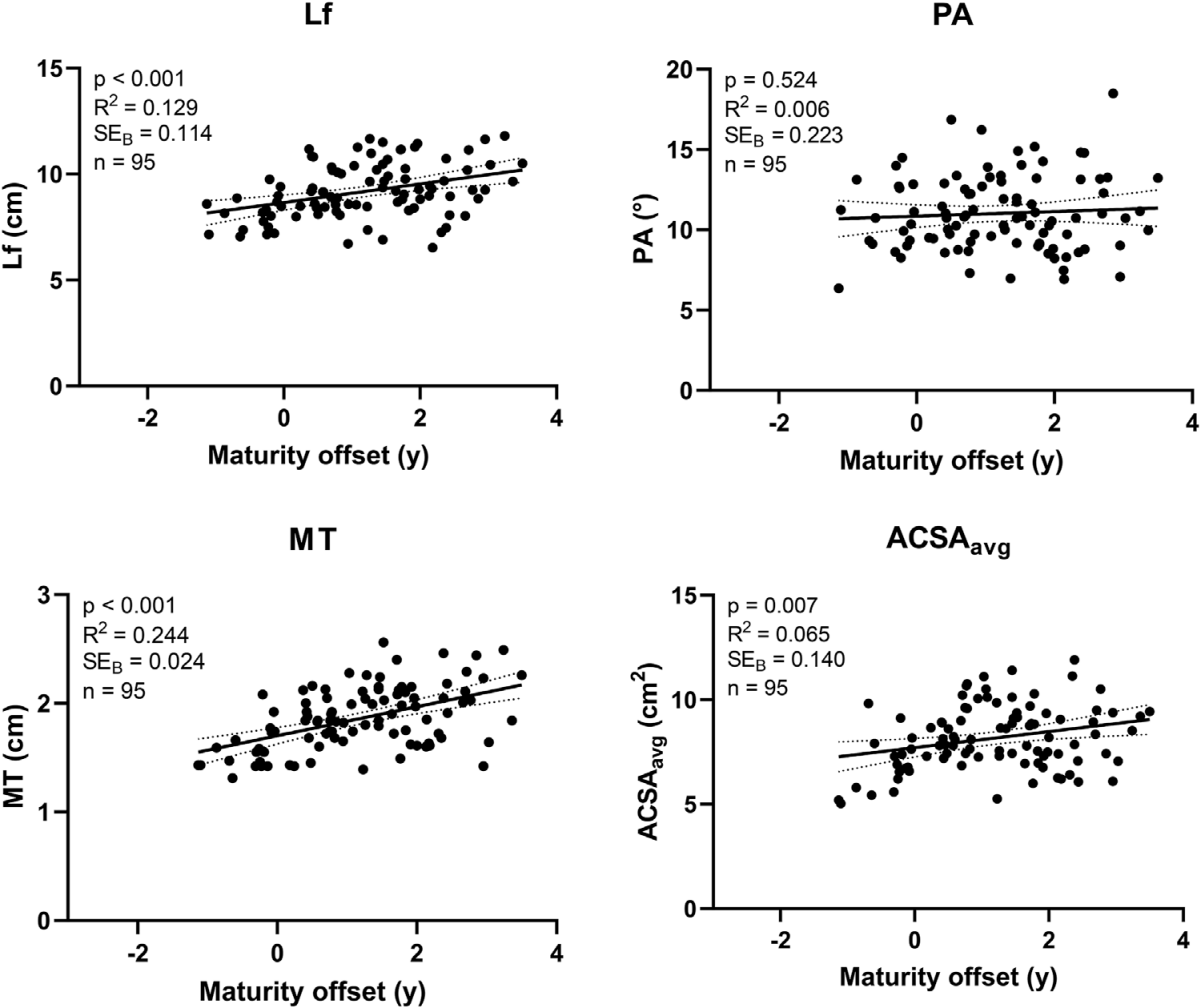
Linear regression analyses assessing the association of variables related to biceps femoris long head morphology with biological maturation (i.e., the maturity offset) in competitive alpine skiers around the growth spurt (i.e., U16 skiers). Lf: fascicle length; PA: pennation angle; MT: muscle thickness; ACSA_avg_: average anatomical cross-sectional area.

Figure 2 shows the results of the linear regression analyses regarding the association of BFlh morphology with maturity offset. Lf (*p* < 0.001), MT (*p* < 0.001) and ACSA_avg_ (*p* = 0.007) were found to be significantly associated with maturity offset. Lf explained 12.9% of the variance in maturity offset (*R* ^2^ = 0.129), while MT and ACSA_avg_ explained 24.4 and 6.5% (*R* ^2^ = 0.244 and *R* ^2^ = 0.065, respectively). There was no significant association of maturity offset with PA (*p* = 0.524).

### Association between biceps femoris long head morphology and traumatic injuries of the lower extremities within the subsequent season

In the subsequent season (i.e., the 12 months after the baseline measurements), a total of 37 of the 95 youth skiers were suffering from traumatic lower extremity injuries, of which 16 sustained traumatic knee injuries (11 sprains, four contusions, one undefined trauma). Other common injuries were ankle sprains (16 affected skiers), as well as contusions or fractures at the lower leg (four skiers affected). A total of 66.7% of the injuries occurred in the first half of the year (i.e., during the competition season period from mid-November to mid-April), and 33.3% of the injuries occurred in the second half of the year (i.e., during the preseason period from mid-April to mid-November). Binary logistic regression analysis revealed a significant association between the predictor ACSA_avg_ and the occurrence of traumatic lower extremity injuries in the upcoming season (Chi-square = 4.627, R_Nagelkerke_^2^ = 0.064, *p* = 0.031, *n* = 95). If ACSA_avg_ increases by one unit (i.e., 1 cm^2^), the relative probability that a youth skier sustains a lower extremity injury decreases by 26.5% (Wald = 4.328, e^B^ = 0.735, *p* = 0.037). The predictors Lf, PA and MT and the potential confounder maturity offset were removed from the model by the backward LR method.

## Discussion

The major findings of this study were as follows: 1) male and female skiers did not differ in BFlh morphology; 2) regardless of sex, older skiers had, on average, longer fascicles; 3) Lf, MT and ACSA_avg_ were significantly associated with maturity offset, but no association was found between maturity offset and PA; and 4) ACSA_avg_ was found to be associated with the occurrence of traumatic lower extremity injuries in youth competitive alpine skiers in the upcoming season.

BFlh Lf, PA, MT and ACSA_avg_ did not differ between male and female skiers. One explanation for this might be found in the higher maturity offset value of the female skiers compared to the male skiers of our cohort: female skiers were on average 1.7 years more advanced in their biological maturation and had already clearly passed their growth spurt, while male skiers were still close to their age at peak height velocity. Thus, differences in BFlh morphology attributable to hormonal-related influences on muscle growth may not be fully detectable at this stage in such comparisons. Similarly, the values for PA, MT and ACSA_avg_ were not significantly different between the U16 and U15 skiers. The only significant age difference was found for the Lf values, where older skiers had on average longer fascicles than younger skiers (9.5 ± 1.3 cm vs 8.9 ± 1.3 cm). Lf adaptations toward longer lengths during growth and maturation have already been shown in several studies, as highlighted in a recent review (Tumkur Anil Kumar et al., 2021). An impressive example that muscle longitudinal growth (i.e., Lf increase) can be affected by bone growth was presented in a case report by (Boakes et al., 2007). The authors investigated the change in Lf and sarcomere length and the number of vastus lateralis muscles in a 16-year-old girl who underwent a bone distraction procedure that lengthened the femur by 10%. The results showed that Lf increased from 9.1 to 19 cm during the distraction phase and then remained stable during the consolidation phase. Thus, it is plausible that growth-related changes leading to an increase in bone length as a result of physiological development may be a major driver of longitudinal muscle hypertrophy, especially in muscles of the lower extremities (Kubo et al., 2001).

Although the age-related differences in Lf in the present study are of small magnitude, they could nevertheless have functional consequences. Animal studies show that muscle fiber length can influence whole muscle maximal unloaded shortening velocity (Spector et al., 1980). In humans, however, studies that have shown relationships between Lf and functional adaptations are still scarce, although it has been shown that Lf is greater in sprinters than in distance runners (Abe et al., 2000) and is related to sprint performance in 100-m sprinters (Kumagai et al., 2000). Another functional consequence of a greater Lf may relate to the joint angle-torque curve. A training intervention based on NHE (i.e., involving lengthening muscle actions (Raiteri et al., 2021)) resulted in an adaptation toward a greater joint angle at which MVT is generated (Brockett et al., 2001).

The average values of Lf, PA and MT (9.2 ± 1.3 cm, 11.0 ± 2.3° and 1.9 ± 0.3 cm) measured across all study participants appear plausible when compared with values from panoramic ultrasound studies, which investigated youth athletes from a different sport (Lacome et al., 2019; Ritsche et al., 2021). Interestingly, when comparing these values with those of adult elite competitive alpine skiers (Lf = 8.1 ± 1.4 cm, PA = 14.9 ± 4.1° and MT = 2.1 ± 0.3 cm) reported in a previous publication from our lab (Franchi et al., 2020b), it seems that youth skiers have on average longer Lf and smaller values of PA and MT. The observation that adult elite skiers show shorter Lf values may be of particular interest because in the present study, Lf increases with age and thus during maturation. Therefore, it seems that, at least for competitive alpine skiers, once the growth spurt is completed, BFlh morphology may change toward shorter Lf and larger PA and MT. Potential explanations for such adaptations could be related to an increase in radial muscle hypertrophy (Jorgenson et al., 2020), possibly due to increased resistance training volumes.

In contrast to chronological age, where age-related differences were only observed for Lf, maturity offset had significant influences on Lf, MT and ACSA_avg_. With increasing maturity offset, all the abovementioned variables of BFlh morphology increased. This was previously shown in pre, circa- and postpeak height velocity school boys, where muscle architecture variables increased from pre-to postintervention (Radnor et al., 2020). The only exception was PA, for which no association with maturity offset was found. In the literature, an increase in PA is described as a consequence of radial muscle fiber hypertrophy as a kind of “packing strategy” (Gollnick et al., 1981). Hypertrophied unipennate muscles have higher PA than untrained muscles (Kawakami et al., 1993). Compared to youth skiers, adult elite skiers (longer exposed to resistance training programs) show greater values of PA (Franchi et al., 2020b). Based on typical coaching concepts, in competitive alpine skiing, the athletic training volume (and thus appropriate resistance training stimuli) increases noticeably after completing the youth level (Läuppi and Spörri, 2014). A possible speculation is that BFlh PA may be specifically dependent on resistance training stimuli, whereas at this age, the variables Lf, MT and ACSA_avg_ typically change in relation to physiological growth alone.

According to the binary logistic regression analysis, an increase in the predictor BFlh ACSA_avg_ by one unit (i.e., 1 cm^2^) decreased the relative probability that a youth skier sustains a lower extremity injury during the upcoming 12 months by 26.5%. This suggests that the ACSA_avg_ of the BFlh mid-belly (i.e., 30–60% of femur length) is a potentially relevant variable in the context of traumatic lower extremity injuries in youth competitive alpine skiers. The injuries occurring in this study mainly included traumatic knee injuries (sprains and contusions), ankle sprains, and contusions or fractures of the lower leg. As already explained in more detail in the introduction section, BFlh stabilizes the knee joint and has the greatest ability to protect the ACL, as it is capable of counteracting internal rotation of the knee, generating large force magnitudes, and opposing the anterior shear force (Maniar et al., 2022). Regarding ankle sprains, the BFlh opposes internal rotation of the tibia (Maniar et al., 2022), which in turn may counteract rearfoot pronation during typical mechanisms leading to ankle sprains (Neumann, 2010). In addition, the BFlh may counteract the hip flexion moment during backward falls with the trunk bent forward, as often occurs in the immediate course of tibial contusions or fractures (Stenroos et al., 2016). Finally, it should be emphasized that ACSA_avg_ appears to play a superior role compared to the muscle architecture variables, as these were removed from the regression model using the LR backward method. ACSA_avg_ can be considered a more global approximation for the overall muscle’s strength capacity (because it covers the ACSA between 30 and 60% of the femur length and thus a large part of the BFlh) and possibly a more clinically relevant structural measure of BFlh than, for example, the local measurements of Lf, PA and MT.

Although conclusions about the functional consequences of differences in BFlh morphology are purely speculative, a larger BFlh ACSA_avg_ could functionally contribute to a higher MVT and RTD. The ability to produce a high MVT and RTD is related to neuronal and muscular factors. It is well accepted that MVT is related to muscle size and that ACSA seems to be an adequate predictor of MVT (Blazevich et al., 2009). Furthermore, it is known that the MVT correlates with the RTD (Mirkov et al., 2004), whereby the correlation increases from the time of force production onset (Andersen and Aagaard, 2006). It is therefore speculated that factors that influence MVT (i.e., ACSA) can also influence RTD (Maffiuletti et al., 2016). Given that the timeframe in which ACL injuries typically occur is less than 60 ms (Bere et al., 2013), it stands to reason that both functional capacities (i.e., MVT and RTD) of the BFlh may be relevant for the prevention of traumatic lower extremity injuries in alpine skiers. In the context of ACL injuries in elite alpine ski racers, for example, Jordan et al. (2015) concluded that the assessment of MVT and RTD of hamstrings and quadriceps muscles are important determinants in a comprehensive strength assessment.

### Study limitations and methodological considerations

The present study has some limitations that one should be aware of when interpreting its findings. First, although around the growth spurt, the maturity offset can be estimated with proven validity using the Mirwald formula, the estimation accuracy decreases with increasing deviation from the 0 point (i.e., the age at peak height velocity) in both positive and negative directions. The maturity offset values collected in the present study tend to be above the zero point and for female ski racers slightly outside the recommended limit of −1 to +1. Second, the data collected via the OSTRC questionnaire were self-reported by the skiers. Thus, the quality of the data strongly depends on the answers provided. To ensure sufficient data quality, skiers were assisted by their parents in answering the prospective surveys and were retrospectively interviewed by an experienced sports physician. Third, the ultrasound-based assessment of muscle morphology and associated manual evaluations are dependent on the operator/evaluators. Adequate training is therefore essential for the measurement and analysis to ensure reliability. A high reliability of the same operators/evaluators who conducted the current study has already been reported in another study and can be assumed to be on the same order of magnitude for the current study (Franchi et al., 2020a; Franchi et al., 2020b). Fourth, given the multifactorial system of injury causation, there may have been some risk of bias from unknown confounders, a circumstance that certainly limits the ability to draw conclusions about cause and effect. Nonetheless, BFlh ACSA_avg_ showed a significant association with traumatic lower extremity injuries and, therefore, can be considered a meaningful proxy measure. Moreover, the experimentally determined relationship between BFlh ACSA_avg_ and traumatic lower extremity injuries is also very plausible from a theoretical/biomechanical point of view, as already outlined above. Fifth, during the 12-months prospective injury surveillance, some dynamic changes in BFlh morphology may have emerged. Thus, by the time of injury, BFlh ACSA_avg_ could have changed and may have slightly differed from the assessment at baseline. However, given the restricted sample size when investigating youth competitive alpine skiers, a certain period of time is required to collect a sufficient number of injury cases to ensure that the study is not underpowered.

## Conclusion

The present study revealed no differences in BFlh morphology between the sexes. However, our results illustrate that in youth competitive alpine skiers, Lf, MT and ACSA_avg_ can be influenced by age and biological maturation. In contrast, no influence on PA was found. In comparison to adult elite alpine skiers (Franchi et al., 2020b), youth skiers in the present study display on average longer Lf but smaller PA and MT. Accordingly, an interesting future research question would be how the resistance training stimulus should be modulated to achieve radial (increase in PA) and longitudinal (increase in Lf) muscle fiber hypertrophy and resulting functional capacities in long-term development. Furthermore, it is worth highlighting that based on the findings of the present study and those of an earlier report (Franchi et al., 2019), biological maturation can influence both the structural (BFlh morphology) and functional dimensions (the measured hamstring peak force value during the execution of NHEs). Finally, the results of the present study further support the important role of the hamstring muscles as a relevant modifiable variable for the purpose of injury prevention, and ACSA_avg_ is a meaningful proxy measure that is associated with the occurrence of traumatic lower extremity injuries.

## Data Availability

The datasets presented in this article are not readily available because their access is restricted to protect the interests of the project partner Swiss-Ski and their athletes. Requests to access the datasets should be directed to joerg.spoerri@balgrist.ch.
